# Age- and Sex-Specific Differences in Multimorbidity Patterns and Temporal Trends on Assessing Hospital Discharge Records in Southwest China: Network-Based Study

**DOI:** 10.2196/27146

**Published:** 2022-02-25

**Authors:** Liya Wang, Hang Qiu, Li Luo, Li Zhou

**Affiliations:** 1 Big Data Research Center University of Electronic Science and Technology of China Chengdu China; 2 School of Computer Science and Engineering University of Electronic Science and Technology of China Chengdu China; 3 Business School Sichuan University Chengdu China; 4 Health Information Center of Sichuan Province Chengdu China

**Keywords:** multimorbidity pattern, temporal trend, network analysis, multimorbidity prevalence, administrative data, longitudinal study, regional research

## Abstract

**Background:**

Multimorbidity represents a global health challenge, which requires a more global understanding of multimorbidity patterns and trends. However, the majority of studies completed to date have often relied on self-reported conditions, and a simultaneous assessment of the entire spectrum of chronic disease co-occurrence, especially in developing regions, has not yet been performed.

**Objective:**

We attempted to provide a multidimensional approach to understand the full spectrum of chronic disease co-occurrence among general inpatients in southwest China, in order to investigate multimorbidity patterns and temporal trends, and assess their age and sex differences.

**Methods:**

We conducted a retrospective cohort analysis based on 8.8 million hospital discharge records of about 5.0 million individuals of all ages from 2015 to 2019 in a megacity in southwest China. We examined all chronic diagnoses using the ICD-10 (International Classification of Diseases, 10th revision) codes at 3 digits and focused on chronic diseases with ≥1% prevalence for each of the age and sex strata, which resulted in a total of 149 and 145 chronic diseases in males and females, respectively. We constructed multimorbidity networks in the general population based on sex and age, and used the cosine index to measure the co-occurrence of chronic diseases. Then, we divided the networks into communities and assessed their temporal trends.

**Results:**

The results showed complex interactions among chronic diseases, with more intensive connections among males and inpatients ≥40 years old. A total of 9 chronic diseases were simultaneously classified as central diseases, hubs, and bursts in the multimorbidity networks. Among them, 5 diseases were common to both males and females, including hypertension, chronic ischemic heart disease, cerebral infarction, other cerebrovascular diseases, and atherosclerosis. The earliest leaps (degree leaps ≥6) appeared at a disorder of glycoprotein metabolism that happened at 25-29 years in males, about 15 years earlier than in females. The number of chronic diseases in the community increased over time, but the new entrants did not replace the root of the community.

**Conclusions:**

Our multimorbidity network analysis identified specific differences in the co-occurrence of chronic diagnoses by sex and age, which could help in the design of clinical interventions for inpatient multimorbidity.

## Introduction

With the recent improvements in clinical interventions, advances in public health, lifestyle changes, and environmental exposures, multimorbidity has been a growing global health challenge [1-3]. Although multimorbidity is widely considered as the norm, not the exception, it still has an inconsistent definition and heterogeneity in methodology, which makes it difficult to gauge its prevalence and pattern in the general population [4-6]. In light of the increased mortality, lower quality of life, and higher utilization of health care services associated with multimorbidity [7-11], a global understanding of the multimorbidity pattern and trend is needed. Although a variety of studies have investigated the patterns of multimorbidity [12-16], most of them were conducted using cross-sectional surveys, which were generally limited either by their small number of self-reported conditions or by a small sample size. Therefore, a multidimensional approach is still needed to understand the full spectrum of multimorbidity networks, time trends, and patterns in age and sex, particularly in developing countries or regions [17].

With the enhancement of the storage capacity and accessibility of electronic information systems, digitized clinical record keeping has made routinely collected administrative data of unprecedented depth and variability available to researchers. This provides an opportunity for the application of network analysis to extract conceptual insights from large and messy data sets [18-20]. Although notable studies are few and mostly carried out in developed countries, they have provided promising findings in human phenotypic multimorbidity networks. For instance, based on the disease history of more than 30 million patients collected from hospital claims, correlations for more than 10,000 comorbid disease pairs were calculated and visualized in a phenotypic disease network [19]. Differences in identified multimorbidity across sex and racial groups were identified through macro analyses at the organ level [21,22]. Additionally, a study in Taiwan constructed an epidemiological disease network and examined its temporal pattern [23]. However, it is difficult to investigate the true extent of multimorbidity associations from these studies because of the differential definition of multimorbidity at the cross-sectional level or over a lifetime period, the difference in the measurement of associations, and the study settings that were mainly dominated by developed countries or regions.

To address these gaps, we performed a retrospective study based on all inpatients living in a megacity in southwest China. We applied the standardized definition and classification system of multimorbidity [24]. Our major aim is to provide a multidimensional approach to understand the complex comorbid relationships among the full spectrum of chronic diseases in general multimorbidity inpatients in southwest China. Furthermore, this study aimed to assess age and sex differences in the multimorbidity pattern and investigate highly correlated communities and their temporal trends.

## Methods

### Overview

**Figure 1:**
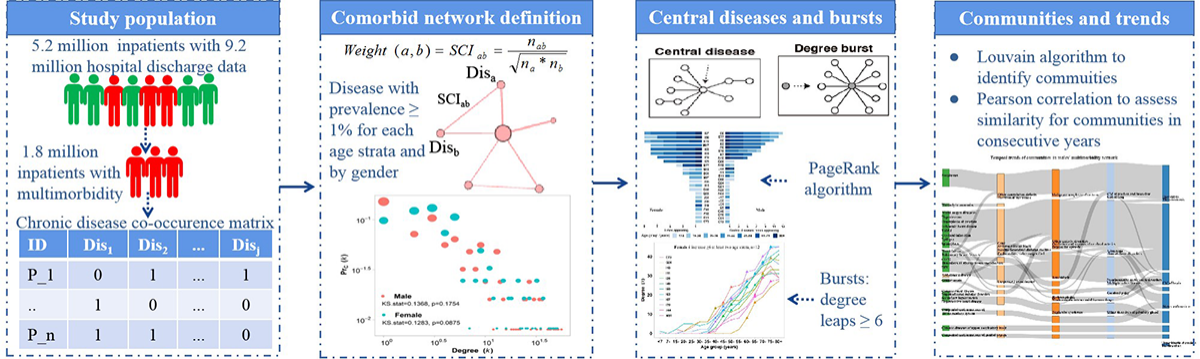
Study workflow. SCI: Salton cosine index. Dis: chronic disease.

The workflow of this study is shown in Figure 1. First, we assessed the quality of the data set and confirmed the study population. Then, the cosine index was selected to construct sex- and age-specific multimorbidity networks. Next, we identified central diseases and bursts, and examined their differences across sex and age. Finally, we divided the networks into communities and assessed their temporal trends. Below, we provide more details on each step of the analysis.

### Ethics Approval

This study was approved by the Ethics Committee of the Health Information Center of Sichuan Province. The data were analyzed anonymously to maintain the privacy of inpatient data. As a study of previously collected administrative data, this work was exempt from informed consent requirements.

### Data Source and Study Design

In this retrospective cohort analysis, we used the regional database of longitudinal clinical data for inpatients, which was provided by the Health Information Center of Sichuan Province. This regional database includes the anonymized hospital discharge reports (HDRs) collected from all of the 534 secondary hospitals and 144 tertiary hospitals in Sichuan Province; therefore, each inpatient’s longitudinal clinical data were available. Each HDR contained information on the anonymized identity, age, sex, residential address, visit and discharge dates, principal discharge diagnosis, and up to 15 secondary diagnoses. All diseases were specified according to the ICD-10 (International Classification of Diseases, 10th revision) codes at 3 digits.

The eligibility criteria included inpatients who were residents of Chengdu and alive for the entire study period. A total of 5.2 million individuals (about 31.5% of Chengdu’s population) with 9.2 million HDRs from 2015 to 2019 were included. As we were interested in diseases (ICD-10: A00-R99), hospitalizations in which the patients were marked only for general symptoms [24] (226,193 cases in total) were removed. According to the sex-specific diagnoses [21,24], 2329 male inpatients and 31 female inpatients were further removed due to conflicts between diagnoses and sex. Finally, the data preprocessing resulted in a total of 8.8 million hospitalizations corresponding to about 5.0 million individuals of all ages, and the sample was large enough to estimate age- and sex-specific multimorbidity patterns.

### Network-Based Analysis

#### Chronic Diseases and Multimorbidity Definition

In 2018, the Academy of Medical Sciences recommended the adoption of a uniform definition and reporting system for multimorbidity [25], which identified multimorbidity as the co-existence of two or more chronic conditions (a physical noncommunicable disease, a mental health condition, or an infectious disease of long duration). Since chronic conditions would not be expected to go away in a single hospitalization period, we considered a 5-year period [13] rather than a single hospitalization for the definition of multimorbidity. The Chronic Condition Indicator [26], developed as part of the Healthcare Cost and Utilization Project, was used to differentiate between acute and chronic ICD-10 codes at 3 digits [24]. A total of 489 and 505 chronic disease codes were separately retained in males and females, respectively.

In order to generate more consistent and reliable estimates, we focused on chronic diseases with ≥1% prevalence for each of the following age strata: <7, 7-14, 15-19, 20-24, 25-29, 30-34, 35-39, 40-44, 45-49, 50-54, 55-59, 60-64, 65-69, 70-74, 75-79, and 80+ years, and for both males and females [20], resulting in a total of 149 and 145 chronic diseases, respectively, which were further used in downstream analyses (Multimedia Appendix 1).

#### Multimorbidity Network Generation and Network Properties Calculation

A multimorbidity network developed from inpatients contains a set of nodes that are connected through edges. The node represents a chronic disease (ICD-10 codes at 3 digits), such that the node size is proportional to the disease prevalence and its color identifies the ICD-10 category.

The edge in the multimorbidity network denotes the comorbid strength between co-existence diseases. Typically, the higher the comorbid strength of a disease pair, the lower the probability of co-existence by chance alone [19,20,27]. The relative risk (RR; calculated in Equation 1) or Pearson correlation coefficient (*ϕ*, calculated in Equation 2) was often used to quantify the comorbid strengths of disease pairs [19,20,27]. These 2 measures are not entirely independent of each other, as they are both affected by the sample size and have intrinsic bias [19]. As we were interested in tightly interconnected disease pairs, mutually exclusive disease pairs with negative comorbid strengths (RR < 1 or *ϕ* < 0) were excluded. Since the Salton cosine index (SCI; calculated in Equation 4) is immune to the sample size and only considers the co-occurrences and the prevalence of multimorbidity [28], we selected it to construct and compare the multimorbidity networks with a widely varied sample size in each of the sex- and age-specific groups.



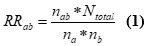





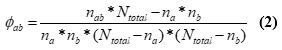





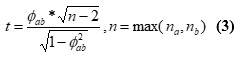





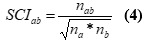



where *n_ab_* denotes the number of co-occurrences of diseases *a* and *b*, *n_a_* and *n_b_* represent the number of occurrences of diseases *a* and *b*, respectively, and *N_total_* is the total number of inpatients in the sex/age-specific group.

**Figure 2:**
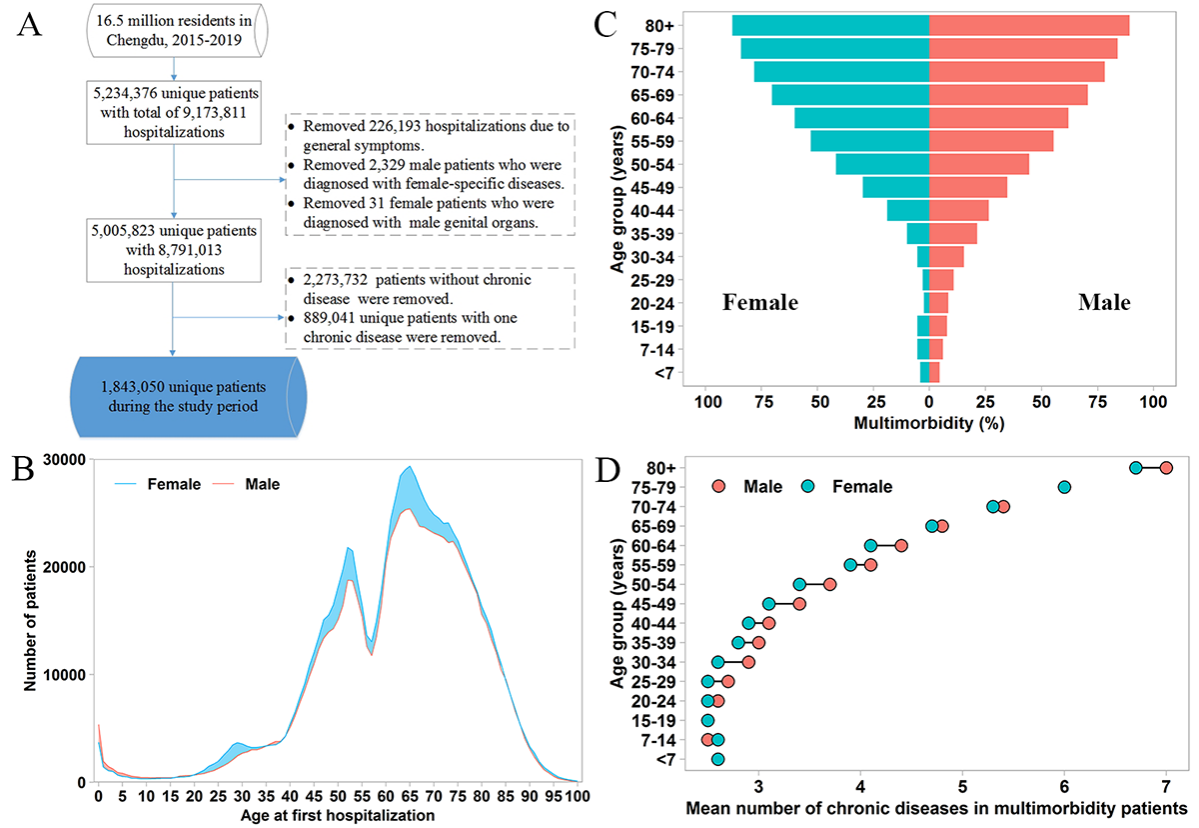
Characteristics of the study population. (A) Selection flow of the study population with multimorbidity. (B) Age and sex distribution for 1.8 million unique inpatients with multimorbidity. (C) Age- and sex-specific percentages of inpatients with multimorbidity among 5.0 million unique inpatients. (D) Age- and sex-specific mean numbers of chronic diseases among 1.8 million unique inpatients with multimorbidity.

Generally, a cutoff for the SCI is defined by assessing the relationship between the Pearson correlation and SCI, where the number of significantly correlated diseases is equal in both networks [29]. For each sex- and age-specific stratum, the steps to find a cutoff for the SCI are as follows: Step 1, Calculate the Pearson correlation coefficient (*ϕ*, calculated in Equation 2) and select the statistically significant correlations at α=.01 (calculated in Equation 3); Step 2, Find the minimum number of disease pairs 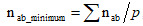, where *p*, the maximum number of edges possible among *n* nodes detected in step 1, is equal to *n*(*n*-1)/2; Step 3, Find the number of pairs (*q*), where *n_ab_* ≥ *n_ab_*__minimum_; Step 4, Find the SCI cutoff (Figure 2B), where the number of pairs is equal to *q*, detected in step 3. The above steps were used to create networks for males and females in each of the 16 age groups and then merge the same edges in different age groups into the general networks for males and females.

The Kolmogorov-Smirnov test was applied to investigate whether the degree distribution follows a power law. The structural properties can be measured using several network indices, such as the density, diameter, average path length, degree, weighted degree, closeness centrality, and betweenness centrality [30]. The closeness centrality measures the shortest distance of the disease away from other chronic diseases. Hence, the higher the closeness centrality of a disease, the higher the risk of co-occurrence with different diseases in fewer number of steps. The betweenness centrality denotes the number of shortest paths through a disease. Then, the higher the betweenness centrality of a disease, the higher the likelihood to form bridges between other diseases.

#### The Central Diseases, Hubs, and Bursts

In order to distinguish the node centrality in the network, the PageRank algorithm [31] was applied, which considers the edge weights. The higher the PageRank value, the more “central” the disease [32]. The parameters were set as commonly assumed, where epsilon = 0.001 and probability = 0.85. Since no established guideline exists for how many nodes are central and since the number of nodes hugely differed among all the age groups in our study, we defined the central diseases as the nodes with the top 10 percentile of the PageRank value across the 16 age strata of males and females.

The connectivity of disease *a* is defined as the sum of all weights of all edges attached to it, which quantifies how strongly a disease is connected to the others. Diseases with larger connectivity are more likely to have a “system-wide” impact on the network. In the study, diseases with the top 10 percentile connectivity values for each of the 16 age strata of males and females have been referred to as the corresponding hubs.

In order to find the nodes with a vastly increased number of edges across age groups (defined as bursts) and to explore the sex difference in the age where the first large leaps occurred, we separately constructed male and female age-based trajectories of degree (*k*) for each node. The nodes with degree leaps ≥6 in the consecutive age groups with such leaps appearing at least one time in the subsequent stratum were defined as bursts. These bursts play essential roles in increasing the multimorbidity burden. Therefore, detecting the age of the first large leaps can help to understand the progression of multimorbidity.

#### Community Detection and Temporal Trends of Communities

Community detection separates the nodes of a generic undirected network into communities, such that connections within communities are stronger than those between them [33,34]. In order to identify distinct clusters of co-occurring diseases, we applied the Louvain method, a heuristic method based on modularity optimization [35]. Modularity *Q* is widely used to compare the partition quality and as an objective function to be optimized [35]. Furthermore, the community detection algorithm used in our analysis considers the weight of the links. Eigenvector centrality measures the influence of a node in a network [36]. The node with the largest eigenvector centrality in the community was therefore considered as the community root.

Observing how communities change over time can also provide valuable information about the network [23]. We applied the same methodology year by year and compared the results across time. As a result, we obtained the temporal trends of the multimorbidity networks. The Pearson correlation coefficient was used to measure the correlation for communities received in consecutive years.

All statistical analyses, network constructions, and visualizations were conducted in R software (version 3.5.1; R Development Core Team).

## Results

### Chronic Diseases and the Prevalence of Multimorbidity

About 5.0 million unique inpatients (representing about 30.3% of the overall Chengdu population) were enrolled in the study, among which 36.8% (a total of 1,843,050 unique inpatients, about 11.2% of the Chengdu population) had two or more chronic diseases (Figure 2). Demographically, the 1.8 million unique inpatients with multimorbidity consisted of inpatients of all ages with a higher percentage of females (52.1%). Generally, males had a statistically higher percentage of multimorbidity compared with females, except for the age group of 70-79 years (as shown in Multimedia Appendix 2). In addition, males in the middle-age (30-34 years and 45-64 years) and older elderly (80+ years) age groups had a large number of chronic diseases compared with females.

### Properties of Age- and Sex-Specific Multimorbidity Networks

**Figure 3:**
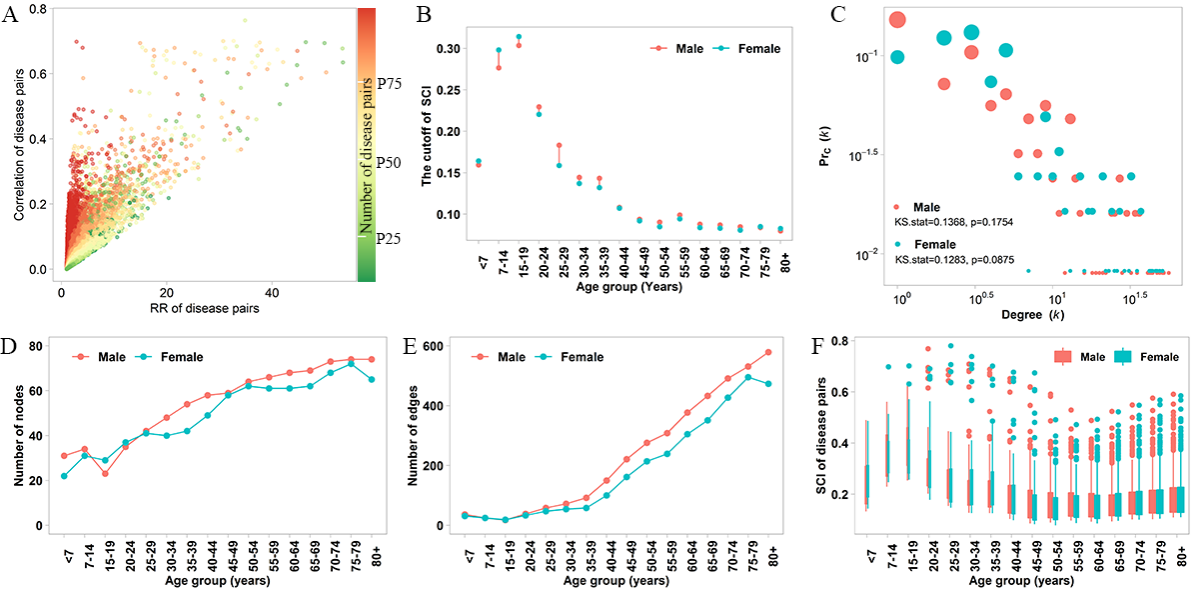
The properties of sex-specific phenotypic multimorbidity networks. (A) Scatter plot between the relative risk (RR) and the Pearson correlation coefficient of disease pairs; due to the interest of tightly interconnected diseases, we excluded mutually exclusive disease pairs with RR <1 or correlation <0. (B) The cutoff of the Sclton cosine index (SCI) where the numbers of significant disease pairs are equal in networks using the Pearson correlation coefficient and SCI. (C) Degree (k) distributions for sex-specific multimorbidity networks using the SCI. (D) and (E) The numbers of connected nodes and edges in each multimorbidity network across age strata and by sex. (F) Box plot of the SCI across age strata and by sex. The width of the box is proportion to the number of edges in each strata’s network.

The phenotypic multimorbidity network analysis identified the network’s global structure and uncovered chronic diseases with a closer co-occurrence (Figure 3). The RR and Pearson correlation coefficient used to measure disease co-occurrence are not entirely independent of each other (Figure 3A). Therefore, the SCI was used to measure the strength of comorbid diseases, and the cutoff of the SCI was determined by assessing the relationship between the Pearson correlation coefficient and SCI (Figure 3B). The cumulative distribution of the number of edges by nodes (degree (*k*) distribution) presented exponential decays (Figure 3C). Both the multimorbidity networks of males and females were scale-free as the distribution followed a power law (Kolmogorov-Smirnov test, *P*=.18 in the male network and *P*=.09 in the female network). The number of nodes and edges for the multimorbidity networks across age strata and by sex ranged from 22 to 74 and 18 to 579, respectively (Figure 3D and 3E). For patients above the age of 30 years, the number of edges we found was more significant in the male multimorbidity network. The number of edges became smaller in the lower age groups, but stronger disease connections were identified (Figure 3F). Table 1 lists the topological properties of each network. Generally, the multimorbidity networks in the younger age groups (≤40 years old) were sparser, except for females <7 years old. The maximum diameter and average path length of the female multimorbidity network were 10 and 4, respectively, which were larger than those of the male network (8 and 2.8, respectively). The average closeness centrality in the multimorbidity network of middle-aged (30-54 years) males was significantly higher than that for females (Wilcoxon test, both *P*<.05).

**Table 1:** Sex- and age-specific multimorbidity network properties.

Network in each age group (years)		Density	Diameter	Average path length	Average degree	Avg.w degree^a^	Avg.clos centrality^b^	Avg.bet centrality^c^
**Male multimorbidity network**								
	<7	0.077	4	1.6	2.3	0.58	0.74	4.4
	7-14	0.045	4	1.5	1.5	0.50	0.82	2.4
	15-19	0.071	4	1.5	1.6	0.62	0.86	3.0
	20-24	0.064	4	1.9	2.2	0.71	0.67	7.3
	25-29	0.067	5	2.4	2.8	0.75	0.57	16.5
	30-34	0.064	8	2.5	3.0	0.73	0.52^d^	23.4
	35-39	0.064	6	2.4	3.4	0.78	0.51^d^	20.4
	40-44	0.091	6	2.8	5.2	1.02	0.40^d^	57.8
	45-49	0.129	6	2.7	7.5^d^	1.30^d^	0.38^d^	63.5
	50-54	0.137	5	2.6	8.6^d^	1.47	0.39^d^	73.6
	55-59	0.144	5	2.5	9.3	1.61	0.41	64.5
	60-64	0.165	5	2.4	11.1	1.84	0.43	62.1
	65-69	0.185	6	2.3	12.6	2.13	0.45	64.0
	70-74	0.187	5	2.1	13.5	2.40	0.48	58.6
	75-79	0.197	5	2.1	14.4	2.63	0.49	61.8
	80+	0.214	4	1.9	15.6	3.00	0.53	50.2
**Female multimorbidity network**								
	<7	0.134	2	1.3	2.8	0.74	0.83	4.0
	7-14	0.054	3	1.4	1.6	0.58	0.82	2.3
	15-19	0.047	2	1.2	1.3	0.50	0.91	1.7
	20-24	0.050	4	1.8	1.8	0.59	0.72	4.6
	25-29	0.057	5	2.1	2.3	0.64	0.61	8.7
	30-34	0.069	8	3.4	2.7	0.70	0.42	45.5
	35-39	0.067	10	4.0	2.8	0.70	0.36	69.2
	40-44	0.085	9	3.7	4.1	0.82	0.34	88.7
	45-49	0.098	6	3.1	5.6	0.93	0.34	94.3
	50-54	0.113	7	2.9	6.9	1.08	0.36	82.2
	55-59	0.131	6	2.7	7.8	1.30	0.39	68.7
	60-64	0.167	5	2.4	10.0	1.65	0.43	59.4
	65-69	0.186	5	2.3	11.3	1.96	0.45	50.2
	70-74	0.187	5	2.2	12.6	2.28	0.48	58.5
	75-79	0.194	5	2.1	13.8	2.57	0.50	57.2
	80+	0.227	4	2.0	14.6	2.80	0.53	47.3

^a^Avg.w degree: average weighted degree.

^b^Avg.clos centrality: average closeness centrality.

^c^Avg.bet centrality: average betweenness centrality.

^d^The values in the multimorbidity network in males were statistically higher than those in females (*P*<.05).

### Age- and Sex-Specific Differences in Central Diseases, Hubs, and Bursts

**Figure 4:**
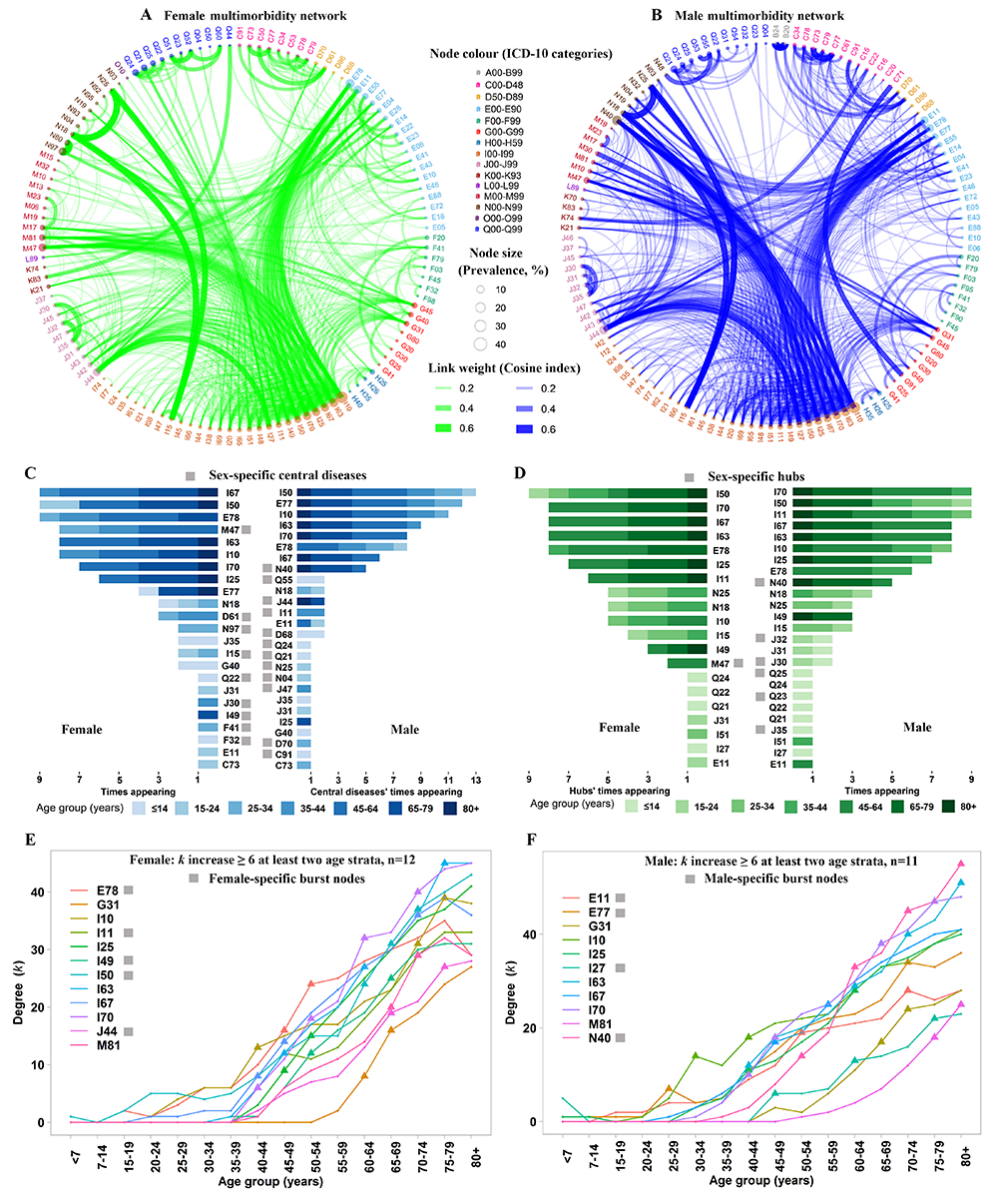
Multimorbidity networks, central diseases, hubs, and connectivity trajectories. Age-adjusted multimorbidity network in females (A) and males (B). Nodes represent chronic diseases (ICD-10 [International Classification of Diseases, 10th revision] codes at 3 digits), such that the node size is proportional to the disease prevalence among multimorbidity patients and its color identifies the ICD-10 category. Link weights are proportional to the magnitudes of the cosine index. (C) The central diseases in each age strata by sex. Diseases with the top 10 percentiles for PageRank in each strata were identified as central diseases. (D) The hubs in each age strata and by sex. Nodes with the top 10 percentiles for hubs in each strata were identified as hubs. The age-based trajectories of the degree (k) of bursts in females (E) and males (F). The triangles indicate degree leaps ≥6 through the consecutive age groups. The disease with at least two such degree leaps was defined as a burst, which means the bursts of disease associations leading to multimorbidity.

The female and male multimorbidity networks are visualized in Figure 4A and 4B, respectively. According to the frequency and comorbidity strength, the top 20 comorbid disease pairs involved 13 diseases, among which 11 diseases (E11, E78, I10, I11, I25, I27, I50, I63, I67, I70, and J44) were common to both males and females, and 2 diseases were sex specific (hyperplasia of the prostate [N40] in males and spondylosis [M47] in females). The most comorbid disease pair was essential hypertension (I10) with cerebral infarction (I63), which occurred in males older than 30 years and females older than 40 years. Notably, there existed a few disease pairs that exhibited strong comorbid strengths but only occurred in a typical age group, for instance, congenital malformation co-existence in children <7 years old. Based on the PageRank algorithm, 23 and 26 chronic diseases were identified as central diseases in the female and male multimorbidity networks. Among these diseases, 14 chronic diseases were common to both males and females, and comprised critical diseases across different ages, such as heart failure (I50), essential hypertension (I10), glycoprotein metabolism disorders (E77), and lipoprotein metabolism disorders (E78) (Figure 4C). Interestingly, depressive episodes (F32) and other anxiety disorders (F41) represented the central diseases among females aged 7-14 years and 25-29 years, respectively. A total of 26 unique diseases were hubs in the multimorbidity networks, including 19 hubs common to both males and females, 1 female-specific hub (spondylosis at 50-59 years), and 6 male-specific hubs (Figure 4D). Furthermore, for each burst, which had at least 2 degree leaps ≥6 through consecutive age groups, connectivity trajectories across age groups are presented in Figure 4E and 4F. A total of 7 burst nodes were common to both males and females, among which essential hypertension (I10) first occurred in men aged 30 to 34 years. Among the 4 male-specific burst nodes, the earliest leaps were glycoprotein metabolism disorders (E77), which happened at 25-29 years. Remarkably, 9 diseases were classified as not only central diseases, but also hubs and bursts. Among them, 5 were common to both males and females, including essential hypertension (I10), chronic ischemic heart disease (I25), cerebral infarction (I63), other cerebrovascular diseases (I67), and atherosclerosis (I70). Therefore, particular diseases act as both bursts for increasing the network complex and as hubs for having a “system-wide” impact on the network, and some of these act as central diseases for playing the most important role in the network.

**Figure 5:**
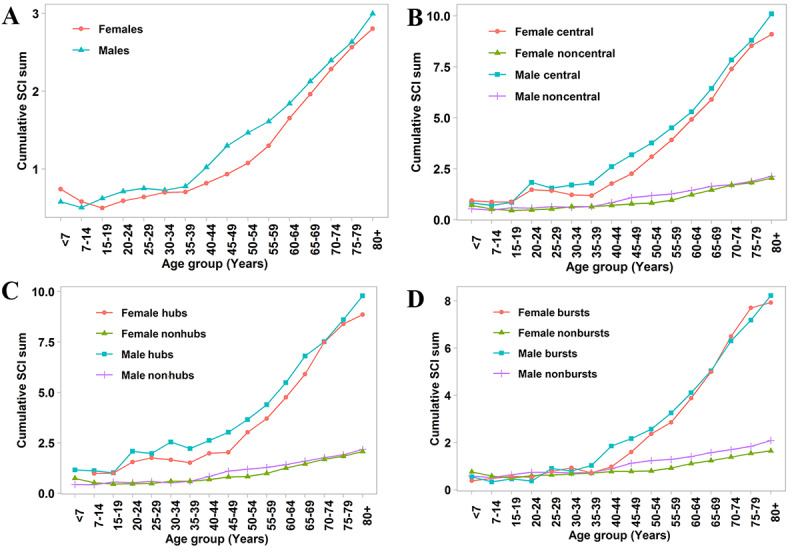
Sex-specific connectivity (cumulative of the node-average Sclton cosine index [SCI]) across age groups. All nodes (A), central diseases vs noncentral diseases (B), hubs vs nonhubs (C), and bursts vs nonbursts (D).

Disease progression by age was evaluated by analyzing the connectivity trajectories of each disease (Figure 5). Males had a higher connectivity, except for the youngest age groups (≤14 years old). In contrast, females had a steeper slope, particularly those aged 55+ years. The central diseases in both males and females showed a higher connectivity compared with noncentral diseases, and the connectivity difference between central diseases and noncentral diseases increased with age among males and females older than 35 years. A similar pattern also appeared in the hubs, but its connectivity difference was more conspicuous at a younger age compared with that in central diseases, which is consistent with the network topology. Subsequently, the bursts in males and females had a higher connectivity after the initial degree leaps ≥6 occurred (males older than 35 years and females older than 40 years).

### Temporal Trends of Communities

**Figure 6:**
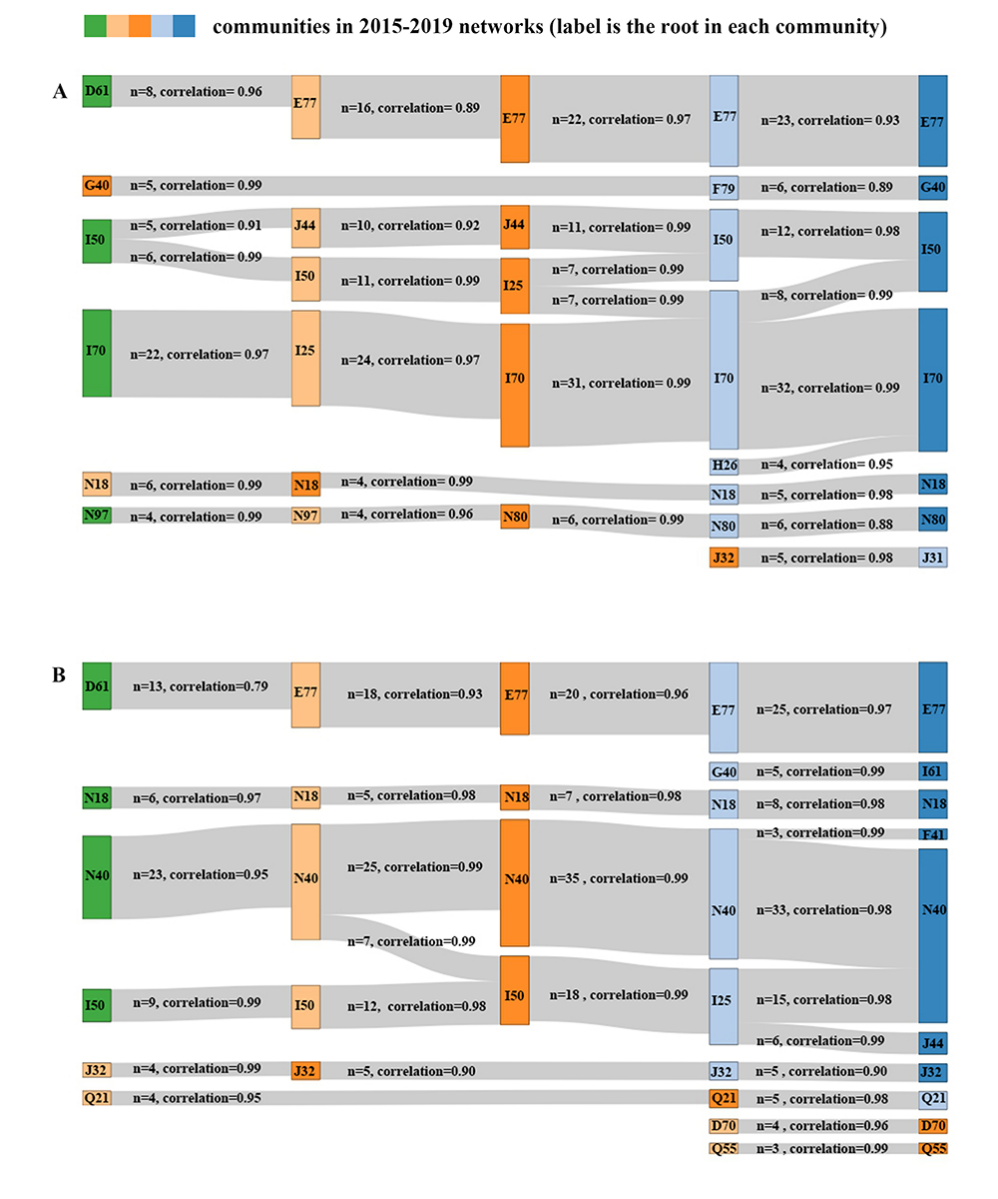
Temporal trends of communities in the female (A) and male (B) multimorbidity networks. By conducting networks year by year and comparing across time, we were able to obtain the temporal trends of the communities. The root, defined as the node with the highest eigenvector centrality within the community, is labeled using ICD-10 (International Classification of Diseases, 10th revision) at 3 digits. Similarity over time is assessed using the Pearson correlation coefficient for communities obtained in consecutive years, and unsignificant (*P*>.05) similarities are excluded. The “n” value is the number of chronic diseases consistent in the consecutive years.

The community structures showed little variation across time, while the community root tended to be stable throughout time (Figure 6). The number of chronic diseases in the community increased over time, consistently in both males and females, and the new entrants did not replace the community root. For instance, the number of diseases in the community, among which the disorder of glycoprotein metabolism (E77) or other aplastic anemias (D61) was identified as the root, increased from 8 to 23 in the female community and from 13 to 25 in the male community. In addition, the community common to both males and females was defined as having the same root within the community, where many diseases were common to both sex groups and few diseases were sex specific. For instance, in the community with chronic renal failure (N18) as the root, both the female and male communities included the same diseases, such as secondary hypertension (I15), chronic nephritic syndrome (N03), nephrotic syndrome (N04), and disorders resulting from impaired renal tubular function (N25), while the male community also included vitamin D deficiency (E55) and the female community also included gout (M10) and systemic lupus erythematosus (M32). The clustering of mental health disorders, including depressive episode (F32), other anxiety disorders (F41), and somatoform disorders (F45), differed by sex. For example, the male community included only mental health disorders, while the female community included mental health disorders and various physical diseases. As for sex-specific diseases, the majority of female-specific diseases were in a separate community, with female infertility (N97) or endometriosis (N80) as the root. As for male-specific diseases, hyperplasia of the prostate (N40) was consistent throughout time, and its eigenvector centrality was even higher than that of atherosclerosis, heart failure, or cerebral infarction.

## Discussion

### Principal Findings

We constructed multimorbidity networks among multimorbidity inpatients (about 11.2% of the Chengdu population) consisting of all ages, which established the connections between chronic diseases in the general hospitalized population from a megacity with 16.5 million residents in southwest China. Multimorbidity affected people of all ages, and their complex interactions were more intensive among males and inpatients ≥40 years old. Notably, in the female multimorbidity network, mental health disorders co-occurred with various mental and physical diseases (eg, metabolic disorders, cardiovascular diseases, and neurodegeneration diseases), among which the co-existence of a depressive episode with other anxiety disorders was detected in 14 age groups of 7-79 years. Moreover, disease connectivity leaps, central diseases, and highly interlinked communities were detected. To the best of our knowledge, this is the first regional study within a developing country that applied regional hospital discharge records rather than self-reported survey data to provide an overview of the prevalence of multimorbidity, obtain multimorbidity patterns, and assess the sex and age differences. Our results demonstrate that the application of network-based algorithms to routinely collected health care data might provide a way to better screen and identify the complex interactions among chronic diseases.

### Multimorbidity Affects People of All Ages

Multimorbidity affects people of all ages, even children who are inpatients (≤14 years old), where 5% of these children have at least two chronic diseases. The findings were in accordance with what was reported in previous studies based on the general population because the prevalence differed by age range, sex, ethnicity, socioeconomic status, and lifestyle, cultural, and health-seeking behaviors [6,13,37-39]. Our results showed that 36.8% of the inpatients living in Chengdu during the 2015-2019 time period had at least two chronic diseases, which is lower than that in the Netherlands (multimorbidity prevalence of 57%) [39], Spain (multimorbidity prevalence of 43.2%) [40], and Canada (multimorbidity prevalence of 53.3%) [6], but higher than that in the United Kingdom (multimorbidity prevalence of 19%) [14], Scotland (multimorbidity prevalence of 31.1%) [41], Singapore (multimorbidity prevalence of 26.2%) [42], Italy (multimorbidity prevalence of 15.3%) [43], and Denmark (multimorbidity prevalence of 21.6%) [44]. A scoping review found a wide range in multimorbidity prevalence in the general population as reported in studies using a large data set, from 15.3% to 68.4% [45]. The reported multimorbidity prevalence is still highly varied due to inconsistent measurements of chronic conditions and multimorbidity [45,46]. Additionally, a study using claims data in Beijing reported that the prevalence of multimorbidity was 51.6% and 81.3% for middle-aged adults (45-59 years) and older adults (≥60 years), respectively [47], which were higher than the respective prevalences of 41.7% and 75.2% in our study. One explanation might be related to the differential study design, since the study in Beijing used both outpatient and inpatient clinical diagnoses for the measurement of multimorbidity. However, the study in Beijing only used 13 most frequently mentioned diseases to measure multimorbidity and the study population was restricted to people who had been employed, which would limit its generalizability to a general population. Therefore, estimating the prevalence of multimorbidity based on a regional database is essential for the design of health care strategies. To the best of our knowledge, this is the first regional study within a developing country to provide an overview of the prevalence of multimorbidity using regional hospital discharge records rather than self-reported survey data. The prevalence of multimorbidity increased with age, which is in line with the findings of previous epidemiological studies that the prevalence of multimorbidity may be increasing, at least in part, because of population aging [5,6,8,41].

### Age and Sex Differences in Multimorbidity Patterns

We identified the multimorbidity patterns in age- and sex-specific inpatient groups, which were comparable with previous studies in developed countries or regions [6,16,20,21]. For instance, Ioakeim-Skoufa et al [16] found associations of respiratory disorders with circulatory diseases, and depression and anxiety with chronic musculoskeletal diseases. In our study, we identified the most frequent and strongest comorbid disease pairs, such as the associations of circulatory diseases with endocrine diseases, diseases of the musculoskeletal system, and respiratory disorders, which co-occurred more frequently than expected by random chance. Our data set consisted of hospitalizations of all ages, and thus contained information about the diseases that are common and specific among age and sex groups. For example, we identified some disease pairs that occurred throughout life (eg, heart failure co-occurring with complications of heart disease and lipoprotein metabolism disorders co-occurring with diabetes mellitus) and some pairs with a stronger comorbid strength but only occurrence among a typical age group (eg, congenital malformation co-existence in children <7 years old). Intuitively, chronic diseases would be expected to co-occur in an individual if their resilience or vulnerability was altered or if they shared a common pattern of influence [48-50]. Thus, as in previous studies assessing the disease trajectory in patients with depression [51] and type 2 diabetes [52], and the general population [24], regional databases collecting HDRs spanning a sufficient time period (generally 10+ years as in the above mentioned studies) will support further studies to explore the potential causal directions among complex correlations. In accordance with previous studies [21,53], we identified a difference in the associations of mental disorders with physical diseases among different sex groups, and generally stronger associations were found in females than in males. This sex difference within mental health multimorbidity may be related to differences in the patient care–seeking behaviors between males and females, as indicated in a previous study that reported on how social factors can discourage males from seeking mental health care [54]. Our findings support the development of interdisciplinary and multidisciplinary treatment strategies for patients with depression or anxiety [53,55,56], since they frequently had physical diseases, such as metabolic disorders, Alzheimer disease, epilepsy, hypertension, chronic ischemic heart disease, heart failure, cerebral infraction, atherosclerosis, gastroesophageal reflux disease, decubitus ulcer, and spondylosis. The data-driven discovery of diseases co-occurring might be useful to generate potential hypotheses for coexisting diseases (eg, sharing the same gene, having common risk factors, and displaying a consistent temporal progression trend [57,58]) and their differences in age and sex (eg, physical, hormonal, and even genetic differences by sex [59,60] and disease progression with age [24]). Additionally, the data-driven discovery of diseases co-occurring, especially based on a complete population with a high-quality health care database, might have impacts on disease management [50].

We found lifelong comorbid disease pairs among multimorbidity inpatients, for example, lipoprotein metabolism disorders (E78) co-occurring with diabetes mellitus (E11) in both males and females ≥15 years old, heart failure (I50) co-occurring with complications of heart disease (I51) in females ≥15 years old, and a depressive episode (F32) co-occurring with other anxiety disorders (F41) in females aged 7-79 years. Congenital malformations, generally the earliest diagnosed diseases in life (ie, pre- or perinatally), had a higher prevalence in multimorbid girls (<7 years) than in boys (<7 years) in our study, especially congenital malformations of the circulatory system (including congenital malformations of the cardiac septa, Q21; congenital malformations of the pulmonary and tricuspid valves, Q22; congenital malformations of the aortic and mitral valves, Q23; other congenital malformations of the heart, Q24; and congenital malformations of the great arteries, Q25). The prevalence of congenital heart disease in girls was higher than in boys [61], and treatment or progression of congenital heart disease could cause complications of heart disease and heart failure [62,63], which may support the finding in our study that heart failure co-occurring with complications of heart disease was earlier in females than in males. Chronic diseases with the earliest connectivity leaps begin at 25-29 years in males, about 15 years earlier than in females. These findings indicate the need to appropriately handle multimorbidity among youth or middle-aged patients [2,64]. Attention and activities are required to prevent such people from entering the multimorbidity category, especially for lipoprotein metabolism disorders, diabetes mellitus, hypertension, heart failure, spondylosis, chronic renal failure, fibrosis and cirrhosis of the liver, and gout. In addition, appropriate guidelines and flexible care management support systems are required across a broader age range.

Within each multimorbidity network, we identified the central disease, which played the most important role in the network (eg, having a large number of comorbid diseases and thus increasing the scale of network, having a relatively smaller number of comorbid diseases but exhibiting stronger comorbid strengths, and playing the role of connectivity to connect those unconnected diseases). In the biomedical scenario, central diseases may be interpreted as those that are more likely to appear in patients with multimorbidity or lead to multimorbidity. Therefore, common causal genes and molecular processes or signaling pathways may be shared among central diseases and their neighbors [27,65]. We found that circulatory diseases and metabolic diseases were the most important diseases among almost all age groups; thus, clinical studies of the identified central diseases may be helpful to improve prevention strategies and health care policies [2]. Notably, younger females should receive more attention on 2 mental health disorders, depressive episodes and other anxiety disorders, which significantly increased the scale of their multimorbidity network. The observations of central diseases could have important implications on the design of health care prevention, such that measurements targeting a specific factor may benefit many related diseases.

Furthermore, we observed that some communities remained stable across time, while others became more extensive due to the occurrence of more diseases. Few studies have observed the temporal trends of networks or communities [23,37]. Jiang et al found that the network structure, connectivity, and module structure varied across time [23]. The work of van Oostrom et al [66] showed that the prevalence of chronic diseases in the general practice registration over the period between 2004 and 2011 increased from 34.9% to 41.8%, and this increase could be only partially explained by the aging of the population. In our study, the community of mental health disorders in the male multimorbidity network consisted of depressive episodes, other anxiety disorders, and somatoform disorders, which seemed independent with physical diseases, while the community involving females additionally included various physical diseases, such as disorders of lipoprotein metabolism and other lipidemias (E78), essential hypertension (I10), hypertensive heart disease (I11), cerebral infarction (I63), atherosclerosis (I70), gastroesophageal reflux disease (K21), and spondylosis (M47). The clustering difference in mental health disorders according to sex might be related with the higher underdiagnosis rate of mental health disorders in males [67] and provide evidence for differential strategies in diagnosis and treatment for males and females. For instance, when 2 diseases are discordant in terms of their pathogenesis (eg, depression co-occurring with cerebrovascular disease in females), they may require separate time-intensive treatment plans [56,68-70]. Additionally, within the community, both concordant and discordant diseases in terms of their pathogenesis were included (eg, a female community with atherosclerosis as the root and including diabetes mellitus, hypertension, cerebrovascular diseases, mental health disorders, and spondylosis), which might lead to very different management needs and treatment strategies [56,68-71]. Communities can describe the interconnections among chronic diseases, with more tight connections between those in the same community. In a further study, it will be of interest to examine the direction of these interconnections or to explore their common risk factors for priority management.

### Strengths and Limitations

The main strengths of this study can be summarized as follows. First, this is the first regional study in developing countries based on a large-scale data set (8.8 million hospital discharge records) to examine multimorbidity patterns and trends, and their differences across age and sex. Additionally, a network-based approach is applied to extract conceptual insights from routinely collected hospital discharge records. The use of this method can be extended to other health care data sets. Lastly, the use of routinely collected administrative data at a regional level is advantageous because the data are uniformity distributed and unbiased, which provides an opportunity to identify the co-occurrence of rare clinical diseases.

This study has some limitations. First, the main limitation of this study is the unavailability of individual-level socioeconomic status, lifestyle, and clinical variables. These factors would play essential roles in understanding the differences among multimorbidity patterns [23]. This limitation is common among studies that use routinely collected health care data sets. Second, the data set that was used did not contain information on the outpatients who were seeking solely outpatient care. Hence, it is vital to interpret our findings in the context of the inpatient population in a developing country. Third, we excluded individuals who were not alive during the study period to obtain a more homogenous study population, which may underestimate the diseases that have high mortality rates. However, it was demonstrated by a previous study that this exclusion criterion did not drastically impact the results [37].

### Conclusions

In this paper, we performed a network-based analysis of 8.8 million hospital discharge records and identified age and sex differences in multimorbidity patterns and the evolution of multimorbidity over time. This longitudinal study provides the first evidence from a developing country that multimorbidity affects people of all ages and their complex interactions are more intensive among males and inpatients ≥40 years old. Mental health disorders were comorbid with more various mental and physical diseases in females than in males. The lifelong comorbid disease pairs, disease connectivity leaps, central diseases, highly interlinked communities, and age- and sex-specific comorbidity patterns detected in the study might provide suggestions for enhancing integrated management in multimorbidity patients. Meanwhile, the network-based approach applied in our study could investigate all the multimorbidity connections at the population level, which could be used within health care data sets in other settings.

## Appendix

Multimedia Appendix 1 Diseases with prevalence ≥1% in males and females.

Multimedia Appendix 2 Multimorbidity prevalence in each age strata and by sex.

## Abbreviations

HDR: hospital discharge report
ICD-10: International Classification of Diseases, 10th revision
RR: relative risk
SCI: Salton cosine index
