# Correction: Age- and Sex-Specific Differences in Multimorbidity Patterns and Temporal Trends on Assessing Hospital Discharge Records in Southwest China: Network-Based Study

**DOI:** 10.2196/39648

**Published:** 2022-06-16

**Authors:** Liya Wang, Hang Qiu, Li Luo, Li Zhou

**Affiliations:** 1 Big Data Research Center University of Electronic Science and Technology of China Chengdu China; 2 School of Computer Science and Engineering, University of Electronic Science and Technology of China Chengdu China; 3 Business School Sichuan University Chengdu China; 4 Health Information Center of Sichuan Province Chengdu China

In “Age- and Sex-Specific Differences in Multimorbidity Patterns and Temporal Trends on Assessing Hospital Discharge Records in Southwest China: Network-Based Study” (J Med Internet Res 2022; 24(2): e27146) the authors noted one error.

In the originally published paper, the denominator of Equation 2 was incorrect and should have been expressed as a square root.

In the corrected version of the paper, the denominator of Equation 2 has been revised to a square root and is provided below. The calculations in the original publication were according to the correct version of Equation 2. The error in Equation 2 did not influence the results of the originally published paper.



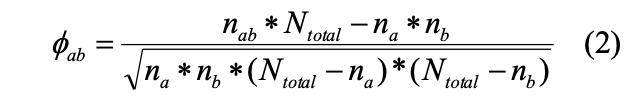



The correction will appear in the online version of the paper on the JMIR Publications website on June 16, 2022, together with the publication of this correction notice. Because this was made after submission to PubMed, PubMed Central, and other full-text repositories, the corrected article has also been resubmitted to those repositories.

