# Supplementary material for: Age- and Sex-Specific Differences in Multimorbidity Patterns and Temporal Trends on Assessing Hospital Discharge Records in Southwest China: Network-Based Study
Source: J Med Internet Res. 2022 Feb 25;24(2):e27146. doi: 10.2196/27146 (PMC8917436; doi:10.2196/27146)
Supplement: Multimedia Appendix 1 [file jmir_v24i2e27146_app1.docx]

**Multimedia Appendix 1.** Diseases with prevalence ≥1% in males and females.

| **Diagnosis Name** | **ICD-10** | **Sex-specific disease ^a^** | **Target disease ^b^** |
| --- | --- | --- | --- |
| Human immunodeficiency virus [HIV] disease resulting in infectious and parasitic diseases | B20 | M & F | M |
| Unspecified human immunodeficiency virus [HIV] disease | B24 | M & F | M |
| Malignant neoplasm of oesophagus | C15 | M & F | M |
| Malignant neoplasm of stomach | C16 | M & F | M |
| Malignant neoplasm of rectum | C20 | M & F | M |
| Malignant neoplasm of liver and intrahepatic bile ducts | C22 | M & F | M |
| Malignant neoplasm of bronchus and lung | C34 | M & F | M & F |
| Malignant neoplasm of breast | C50 | M & F | F |
| Malignant neoplasm of cervix uteri | C53 | F | F |
| Malignant neoplasm of ovary | C56 | F | F |
| Malignant neoplasm of prostate | C61 | M | M |
| Malignant neoplasm of brain | C71 | M & F | M & F |
| Malignant neoplasm of thyroid gland | C73 | M & F | M & F |
| Secondary and unspecified malignant neoplasm of lymph nodes | C77 | M & F | M & F |
| Secondary malignant neoplasm of respiratory and digestive organs | C78 | M & F | M & F |
| Secondary malignant neoplasm of other sites | C79 | M & F | M & F |
| Lymphoid leukaemia | C91 | M & F | M & F |
| Myeloid leukaemia | C92 | M & F | F |
| Anaemia due to enzyme disorders | D55 | M & F | M |
| Thalassaemia | D56 | M & F | M & F |
| Other aplastic anaemias | D61 | M & F | M & F |
| Other coagulation defects | D68 | M & F | M & F |
| Agranulocytosis | D70 | M & F | M & F |
| Immunodeficiency with predominantly antibody defects | D80 | M & F | M & F |
| Other immunodeficiencies | D84 | M & F | M & F |
| Sarcoidosis | D86 | M & F | M & F |
| Subclinical iodine-deficiency hypothyroidism | E02 | M & F | F |
| Other nontoxic goitre | E04 | M & F | M & F |
| Thyrotoxicosis [hyperthyroidism] | E05 | M & F | M & F |
| Thyroiditis | E06 | M & F | M & F |
| Insulin-dependent diabetes mellitus | E10 | M & F | M & F |
| Non-insulin-dependent diabetes mellitus | E11 | M & F | M & F |
| Unspecified diabetes mellitus | E14 | M & F | M & F |
| Other disorders of pancreatic internal secretion | E16 | M & F | M & F |
| Hyperparathyroidism and other disorders of parathyroid gland | E21 | M & F | M |
| Hyperfunction of pituitary gland | E22 | M & F | F |
| Hypofunction and other disorders of pituitary gland | E23 | M & F | M & F |
| Ovarian dysfunction | E28 | F | F |
| Nutritional marasmus | E41 | M & F | M & F |
| Unspecified severe protein-energy malnutrition | E43 | M & F | M & F |
| Protein-energy malnutrition of moderate and mild degree | E44 | M & F | F |
| Unspecified protein-energy malnutrition | E46 | M & F | M & F |
| Vitamin D deficiency | E55 | M & F | M & F |
| Other disorders of amino-acid metabolism | E72 | M & F | M & F |
| Other disorders of carbohydrate metabolism | E74 | M & F | M & F |
| Disorders of glycoprotein metabolism | E77 | M & F | M & F |
| Disorders of lipoprotein metabolism and other lipidaemias | E78 | M & F | M & F |
| Disorders of porphyrin and bilirubin metabolism | E80 | M & F | M |
| Other metabolic disorders | E88 | M & F | M & F |
| Unspecified dementia | F03 | M & F | M & F |
| Schizophrenia | F20 | M & F | M & F |
| Bipolar affective disorder | F31 | M & F | M & F |
| Depressive episode | F32 | M & F | M & F |
| Recurrent depressive disorder | F33 | M & F | F |
| Persistent mood [affective] disorders | F34 | M & F | F |
| Other anxiety disorders | F41 | M & F | M & F |
| Obsessive-compulsive disorder | F42 | M & F | M & F |
| Reaction to severe stress, and adjustment disorders | F43 | M & F | M & F |
| Dissociative [conversion] disorders | F44 | M & F | F |
| Somatoform disorders | F45 | M & F | M & F |
| Eating disorders | F50 | M & F | F |
| Specific personality disorders | F60 | M & F | F |
| Unspecified mental retardation | F79 | M & F | M & F |
| Mixed specific developmental disorders | F83 | M & F | M & F |
| Pervasive developmental disorders | F84 | M & F | M |
| Hyperkinetic disorders | F90 | M & F | M |
| Tic disorders | F95 | M & F | M |
| Other behavioural and emotional disorders with onset usually occurring in childhood and adolescence | F98 | M & F | F |
| Mental disorder, not otherwise specified | F99 | M & F | F |
| Parkinson's disease | G20 | M & F | M & F |
| Other extrapyramidal and movement disorders | G25 | M & F | M & F |
| Alzheimer's disease | G30 | M & F | M & F |
| Other degenerative diseases of nervous system, not elsewhere classified | G31 | M & F | M & F |
| Epilepsy | G40 | M & F | M & F |
| Status epilepticus | G41 | M & F | M & F |
| Migraine | G43 | M & F | F |
| Transient cerebral ischaemic attacks and related syndromes | G45 | M & F | M & F |
| Cerebral palsy | G80 | M & F | M & F |
| Hydrocephalus | G91 | M & F | M & F |
| Senile cataract | H25 | M & F | M & F |
| Other cataract | H26 | M & F | M & F |
| Other disorders of lens | H27 | M & F | M |
| Other retinal disorders | H35 | M & F | M & F |
| Glaucoma | H40 | M & F | M |
| Conductive and sensorineural hearing loss | H90 | M & F | M |
| Multiple valve diseases | I08 | M & F | M & F |
| Essential (primary) hypertension | I10 | M & F | M & F |
| Hypertensive heart disease | I11 | M & F | M & F |
| Secondary hypertension | I15 | M & F | M & F |
| Angina pectoris | I20 | M & F | M & F |
| Acute myocardial infarction | I21 | M & F | M & F |
| Other acute ischaemic heart diseases | I24 | M & F | M & F |
| Chronic ischaemic heart disease | I25 | M & F | M & F |
| Other pulmonary heart diseases | I27 | M & F | M & F |
| Nonrheumatic aortic valve disorders | I35 | M & F | M & F |
| Endocarditis, valve unspecified | I38 | M & F | M & F |
| Cardiomyopathy | I42 | M & F | M |
| Atrioventricular and left bundle-branch block | I44 | M & F | M & F |
| Other conduction disorders | I45 | M & F | M & F |
| Paroxysmal tachycardia | I47 | M & F | M & F |
| Atrial fibrillation and flutter | I48 | M & F | M & F |
| Other cardiac arrhythmias | I49 | M & F | M & F |
| Heart failure | I50 | M & F | M & F |
| Complications and ill-defined descriptions of heart disease | I51 | M & F | M & F |
| Intracerebral haemorrhage | I61 | M & F | M & F |
| Other nontraumatic intracranial haemorrhage | I62 | M & F | M |
| Cerebral infarction | I63 | M & F | M & F |
| Occlusion and stenosis of precerebral arteries, not resulting in cerebral infarction | I65 | M & F | M & F |
| Occlusion and stenosis of cerebral arteries, not resulting in cerebral infarction | I66 | M & F | M & F |
| Other cerebrovascular diseases | I67 | M & F | M & F |
| Sequelae of cerebrovascular disease | I69 | M & F | M & F |
| Atherosclerosis | I70 | M & F | M & F |
| Aortic aneurysm and dissection | I71 | M & F | M |
| Arterial embolism and thrombosis | I74 | M & F | M |
| Other disorders of arteries and arterioles | I77 | M & F | M |
| Vasomotor and allergic rhinitis | J30 | M & F | M & F |
| Chronic rhinitis, nasopharyngitis and pharyngitis | J31 | M & F | M & F |
| Chronic sinusitis | J32 | M & F | M & F |
| Chronic diseases of tonsils and adenoids | J35 | M & F | M & F |
| Chronic laryngitis and laryngotracheitis | J37 | M & F | M & F |
| Unspecified chronic bronchitis | J42 | M & F | M & F |
| Emphysema | J43 | M & F | M & F |
| Other chronic obstructive pulmonary disease | J44 | M & F | M & F |
| Asthma | J45 | M & F | M & F |
| Status asthmaticus | J46 | M & F | M |
| Bronchiectasis | J47 | M & F | M & F |
| Gastro-oesophageal reflux disease | K21 | M & F | M & F |
| Alcoholic liver disease | K70 | M & F | M |
| Fibrosis and cirrhosis of liver | K74 | M & F | M & F |
| Other diseases of biliary tract | K83 | M & F | M & F |
| Psoriasis | L40 | M & F | M |
| Decubitus ulcer | L89 | M & F | M & F |
| Other rheumatoid arthritis | M06 | M & F | F |
| Juvenile arthritis | M08 | M & F | M |
| Gout | M10 | M & F | M & F |
| Other arthritis | M13 | M & F | F |
| Polyarthrosis | M15 | M & F | F |
| Gonarthrosis [arthrosis of knee] | M17 | M & F | M & F |
| Internal derangement of knee | M23 | M & F | M & F |
| Polyarteritis nodosa and related conditions | M30 | M & F | M & F |
| Systemic lupus erythematosus | M32 | M & F | F |
| Scoliosis | M41 | M & F | M & F |
| Other inflammatory spondylopathies | M46 | M & F | F |
| Spondylosis | M47 | M & F | M & F |
| Osteoporosis without pathological fracture | M81 | M & F | M & F |
| Osteonecrosis | M87 | M & F | M |
| Recurrent and persistent haematuria | N02 | M & F | M & F |
| Chronic nephritic syndrome | N03 | M & F | M & F |
| Nephrotic syndrome | N04 | M & F | M & F |
| Chronic renal failure | N18 | M & F | M & F |
| Unspecified renal failure | N19 | M & F | M & F |
| Disorders resulting from impaired renal tubular function | N25 | M & F | M & F |
| Other disorders of bladder | N32 | M & F | M |
| Hyperplasia of prostate | N40 | M | M |
| Other disorders of penis | N48 | M | M |
| Endometriosis | N80 | F | F |
| Absent, scanty and rare menstruation | N91 | F | F |
| Excessive, frequent and irregular menstruation | N92 | F | F |
| Other abnormal uterine and vaginal bleeding | N93 | F | F |
| Menopausal and other perimenopausal disorders | N95 | F | F |
| F infertility | N97 | F | F |
| Other congenital malformations of brain | Q04 | M & F | M & F |
| Other congenital malformations of ear | Q17 | M & F | M |
| Congenital malformations of cardiac septa | Q21 | M & F | M & F |
| Congenital malformations of pulmonary and tricuspid valves | Q22 | M & F | M & F |
| Congenital malformations of aortic and mitral valves | Q23 | M & F | M & F |
| Other congenital malformations of heart | Q24 | M & F | M & F |
| Congenital malformations of great arteries | Q25 | M & F | M & F |
| Congenital malformations of larynx | Q31 | M & F | M & F |
| Congenital malformations of trachea and bronchus | Q32 | M & F | M & F |
| Congenital malformations of lung | Q33 | M & F | M |
| Other congenital malformations of intestine | Q43 | M & F | F |
| Congenital malformations of uterus and cervix | Q51 | F | F |
| Other congenital malformations of F genitalia | Q52 | F | F |
| Undescended testicle | Q53 | M | M |
| Hypospadias | Q54 | M | M |
| Other congenital malformations of M genital organs | Q55 | M | M |
| Polydactyly | Q69 | M & F | M |
| Congenital malformations of the musculoskeletal system, not elsewhere classified | Q79 | M & F | M & F |
| Other congenital malformations, not elsewhere classified | Q89 | M & F | F |

a: ICD-10 codes and sex specificity, M means ICD-10 code is male-specific, F means ICD-10 code is female-specific, M & F means ICD-10 code is common to both males and females; b: chronic diseases with ≥1% prevalence for each of the following age strata: <7, 7-14, 15-19, 20-24, 25-29, 30-34, 35-39, 40-44, 45-49, 50-54, 55-59, 60-64, 65-69, 70-74, 75-79, 80+ years, and for both males and females.
