# Supplementary material for: Age- and Sex-Specific Differences in Multimorbidity Patterns and Temporal Trends on Assessing Hospital Discharge Records in Southwest China: Network-Based Study
Source: J Med Internet Res. 2022 Feb 25;24(2):e27146. doi: 10.2196/27146 (PMC8917436; doi:10.2196/27146)
Supplement: Multimedia Appendix 2 [file jmir_v24i2e27146_app2.docx]

**Multimedia Appendix 2.** Multimorbidity prevalence in each age strata and by sex.

| **Age (Years)** | **Male** | |  | **Female** | |
| --- | --- | --- | --- | --- | --- |
|  | **No. inpatients** | **Multimorbidity prevalence % (95% CI)** |  | **No. inpatients** | **Multimorbidity prevalence % (95% CI)** |
| <7 | 261501 | 4.6 (4.5, 4.7) |  | 207313 | 4.1 (4.0, 4.2) |
| 7-14 | 66009 | 5.9 (5.7, 6.1) |  | 52651 | 5.5 (5.3, 5.7) |
| 15-19 | 32119 | 7.9 (7.6, 8.2) |  | 40848 | 5.4 (5.2, 5.6) |
| 20-24 | 47034 | 8.3 (8.1, 8.6) |  | 190919 | 2.5 (2.4, 2.6) |
| 25-29 | 74490 | 10.8 (10.6, 11.0) |  | 395147 | 3.1 (3.1, 3.2) |
| 30-34 | 91112 | 15.4 (15.1, 15.6) |  | 308542 | 5.4 (5.3, 5.5) |
| 35-39 | 85346 | 21.2 (20.9, 21.4) |  | 174664 | 10.1 (10.0, 10.2) |
| 40-44 | 122381 | 26.5 (26.3, 26.8) |  | 179107 | 18.8 (18.6, 19.0) |
| 45-49 | 176097 | 34.8 (34.6, 35.0) |  | 226198 | 29.7 (29.6, 29.9) |
| 50-54 | 185886 | 44.5 (44.3, 44.7) |  | 228193 | 41.8 (41.6, 42.0) |
| 55-59 | 124735 | 55.3 (55.0, 55.6) |  | 145135 | 53.0 (52.7, 53.3) |
| 60-64 | 175530 | 62.0 (61.8, 62.2) |  | 195506 | 60.2 (60.0, 60.4) |
| 65-69 | 172246 | 70.6 (70.4, 70.8) |  | 198568 | 70.2 (70.0, 70.4) |
| 70-74 | 143662 | 78.2 (78.0, 78.4) |  | 154009 | 78.2 (78.0, 78.4) |
| 75-79 | 121707 | 83.9 (83.7, 84.1) |  | 126687 | 84.0 (83.8, 84.2) |
| 80+ | 145751 | 89.3 (89.1, 89.5) |  | 156730 | 87.9 (87.8, 88.1) |

No. Inpatients: number of inpatients; CI: confidence interval.
